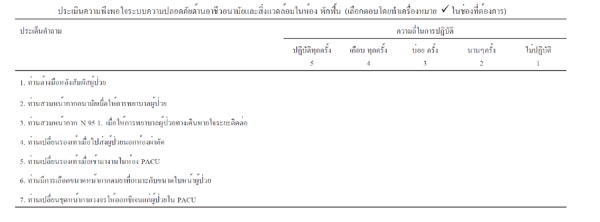# Air Quality and Employee Hygiene-related Behavior in a Post Anesthesia Care Unit in Thailand

**DOI:** 10.31372/20190401.1005

**Published:** 2019

**Authors:** Somphorn Kampan

**Affiliations:** a Anesthesia Nursing Unit, Rajavithi Hospital, Bangkok, Thailand

**Keywords:** PACU, anesthesiology, OR, occupational health and safety, airborne contamination

## Abstract

Post anesthesia care units (PACU) are sanitary spaces at hospitals. Bacterial and fungal contaminants in ambient air can pose significant threats to patient recovery. Excess waste anesthetic gases such as nitrous oxide and desflurane can also pose reproductive, genetic, and other health risks to PACU staff who suffer longterm exposure. Healthcare institutions routinely monitor and study PACU air quality as required by occupational health and safety acts and related regulations, and professional standards of care. This study presents recent data from a PACU intervention at Rajavithi Hospital in Bangkok, Thailand. Rajavithi took measurements of concentrations of airborne fungi, bacteria, desflurane, and nitrous oxide before and after installation of a new ventilation system. Concurrently, the hospital surveyed employees before and after a hazard communication and conducted a training campaign in efforts to understand employee attitudes toward health and safety procedures, and to increase their perceived importance of several PACU-specific protocols. Results showed bacterial contamination fell from 1,307 CFU/m^3^ to 182 CFU/m^3^, fungi fell from 70.4 CFU/m^3^ to 35.8 CFU/m^3^, desflurane fell from 0.25 ppm to 0.21 ppm, and nitrous oxide fell from 21.86 ppm to 20.47 ppm during the intervention while PACU worker attitudes toward health and safety improved. Additional monitoring, communication, and training are recommended for Rajavithi and other healthcare institutions.

## Introduction

In the 1840s, surgeons started using inhalation anesthesia that made it possible for patients to endure extremely painful procedures. Anesthesia use prompted rapid advancements in surgical techniques, but as one problem was solved, another was created. Patients frequently survived invasive procedures in the operating room (OR) only to fall victim to a post-anesthesia complications. High rates of post-surgical fatal and non-fatal complications necessitated a special recovery area near the OR. Florence Nightingale was a pioneer in the post-anesthesia care unit concept in the 1860s, but mainstream hospitals did not adopt the units as a part of standard practice until the 1940s (Cook, 2017).

The European Society of Anesthesiology (Vimlati, Gilsanz, & Goldik, 2009) defines a post anesthesia care unit (PACU) as, “a unit located as close to operating theatres as possible in order to avoid unnecessary time loss for transfer of unstable patients, staffed and equipped for serving for treatment and care of patients during their immediate post anesthesia or post-surgery period, regardless of time of interventions, before they are scheduled to be admitted to general wards, other units of the hospitals or discharged home.” As their utility is well-documented, PACUs are standard facilities in modern hospitals. However, their mere deployment alone does not guarantee a complication-free recovery. As many as 40% of all hospital complications occur in the post-operative environment (Cook, 2017).

### Risks and Hazards of Contamination

Contamination of a PACU can lead to complications in patients and adverse health consequences among hospital workers. Microorganisms such as bacteria and fungi pose serious threats to patients while long-term exposure to anesthesia gases like nitrous oxide and desflurane present significant risks to the health of nurses and anesthetists. Numerous academic, institutional, and governmental studies have demonstrated types of risks poor air quality can pose in PACUs (Caggiano et al., 2014; McGregor, Senjem, & Mazze, 1999; Vimlati et al., 2009).

Post anesthesia care presents significant challenges to hospitals and their people as the health and safety of both patients and hospital workers can be at risk if conditions are not properly maintained. The United States’ National Institute for Occupational Safety and Health [NIOSH] (2007) and Occupational Safety and Health Administration [OSHA] (2000) reported on the potential harm of long-term exposure to anesthesia gases among OR workers. Nitrous oxide (N_2_O) and halogenated gases may leak from a patient’s breathing circuit or they may be exhaled by patients recovering from anesthesia. NIOSH did not give conclusive argument that long-term exposure to N_2_O and halogenated gases like desflurane causes harm, but it made mention of the fact that several studies have linked such exposure to “miscarriages, genetic damage, and concern among operating-room workers.” While OSHA (2000) has yet to set permissible limits for N_2_O and halogenated gases in PACUs, it has provided guidance to other organizations, which developed recommended exposure limits (RELs).

### Regulatory Framework

NIOSH (1977) issued RELs for N_2_O (25 ppm) and some halogenated agents (2 ppm). Other professional organizations offered N_2_O RELs of 50 ppm. Italy, Sweden, Denmark, and the United Kingdom set N_2_O RELs of 100 ppm (McGlothlin, Moenning, & Cole, 2014). OSHA (2000) reported acceptable tolerances for halothane at 50 ppm, and 75 ppm for enflurane. Some relevant publications rely on the 2 ppm RELs for all halogenated agents (e.g., Sessler & Badgwell, 1998), including desflurane even though desflurane was introduced long after NIOSH introduced the 2 ppm standard. NIOSH (2006) reported on the need for desflurane-specific RELs. Aragones et al. (2016) reported desflurane RELs of 5 ppm in Denmark, 10 ppm in Sweden and Poland, and 20 ppm in Austria. There is no consensus on REL for desflurane; NIOSH has not yet issued RELs for desflurane, and few studies have dealt with desflurane—one of the three halogenated agents most often used today, and the halogenated agent measured in this study at Rajavithi Hospital in Bangkok.

Few governments around the world have offered public guidance on safety limits for bacteria and fungi though studies singularly conclude that such contaminants contribute to postoperative complications, especially in patients with diabetes, weakened immune systems, and respiratory diseases (Caggiano et al., 2014; Tang & Wan, 2013). Western Australia Department of Health [WADH] (2015) set a limit of 10 bacterial or fungal colony forming units (CFUs) per cubic meter at patient level. UKDH (2007) recommended bacterial and/or fungal limits of 10 CFU/m^3^ within 300 mm of a wound, and 180 CFU/m^3^ for active OR ambient air. Other countries have set limits of 50–150 CFU/m^3^ for bacteria and fungi (Anil, 2008). Luksamijarulkul and Pipitsangjan (2015) reported an acceptable threshold of airborne bacterial and/or fungal contamination in a general office environment of 500 CFU/m^3^, which may suffice for the lobby of the hospital but not a PACU.

The European Agency for Health and Safety at Work [EU-OSHA] (2014) mentioned waste anesthetic gases posed a “relatively high” risk to healthcare workers. According to EU-OSHA, communication with employees regarding health and safety issues like waste gas exposure is vital to both employer and employee. In the EU-OSHA magazine, Morsing (2011) found inadequate training is the top threat to employee health and safety. Morsing mentioned that because healthcare institutions often have many, varied, and complex machines, they must frequently develop their own understanding of risks and develop plans of action independently to satisfy their unique needs; employee engagement and feedback are critical to continuing success and improvement.

OSHA (2012) regulations grant employees the right to know about chemical hazards in their workplace. In the United States, employers must also instruct employees on how to protect themselves against those hazards. The State of Minnesota’s OSHA (MN-OSHA) implements federal guidelines in three main parts—identification and labelling of hazardous substances, and employee training. MN-OSHA (2017) requires the hazard communication (HazCom) program to inform employees of hazardous substances; records must be retained; employee training must be frequent and documented. Similarly, section 32 of Thailand’s Occupational Safety, Health, and Environment Act (2011) requires employers to conduct hazard assessments, study potential impacts of the working environment on employees, and prepare plans regarding occupational safety, health, and environment for both employees and supervisors.

### Research Aims & Significance

Considering results of historical research, especially publications from governmental organizations (i.e., NIOSH, 2007; OSHA, 2000; UK Department of Health [UKDH], 2007; WADH, 2015), Rajavithi undertook the project certain that installation of a new ventilation system would reduce airborne contaminants in the PACU. The research documents change in air quality in the PACU, and in doing so helps the hospital quantify the value of its capital investment. Legal and professional standards compel the hospital to continually make assessments, monitor data, train and retrain staff in attempts to improve the quality of care and safety in the working environment. As part of broader initiatives to improve the hospital, Rajavithi deployed a HazCom and training campaign and sought to estimate its impacts on PACU employees. Once again, hospital staff expected the campaign to produce positive results. The research documents change in PACU staff attitudes toward health and safety procedures, which helps Rajavithi and other healthcare professionals develop continued training and engagement programs as required by law, ethical codes, or professional standards.

Research regarding air quality in PACUs is available but not in abundance. PACUs are specialized niches of hospital systems, and they receive less attention than other domains in healthcare research. Publication concerning PACU standards and practices in Thailand is rare, which lends importance to this study. Research regarding performance of a HazCom and training program is integral in a healthcare provider’s effort to raise standards in patient care alongside improved occupational health and safety. Thus, stakeholders in Rajavithi Hospital, other hospitals in Thailand, and those around the world may benefit from issues discussed herein and any future studies on related matters.

The utility of the ventilation system is determined by the level to which such systems reduce airborne contaminants (i.e., nitrous oxide, desflurane, bacteria, and fungi). Success of the HazCom and training program is primarily qualified by increases in perceived importance of health and safety behaviors among relevant hospital workers, and secondarily by decreases in airborne contaminants. The researcher assumes that demonstrated improvements in the Rajavithi Hospital PACU implies that other hospitals in Thailand, and elsewhere, could benefit from implementing either or both ventilation systems and campaigns. Ultimately, positive results in this study could result in saving the lives of patients; better air quality and attentive staff will certainly aid in their recovery and improve working conditions for nurses and anesthetists.

## Method

The research was comprised of two main parts—quantitative measurements of airborne pollutants in the Rajavithi PACU, and self-reporting surveys of PACU workers. Each part had two stages: before and after installation of a ventilation system which occurs during a HazCom and training program. Considering the potentially harmful effects to both patients and hospital workers that atmospheric pollution in the PACU can present, this research sought to quantify levels of four separate contaminants—bacteria, fungi, desflurane, and nitrous oxide.

The study then collected survey data to assess the impact of an informational campaign among PACU workers. Two surveys were disseminated—one before launch of the campaign and one following the campaign—to quantify PACU workers’ perceived importance of health and safety procedures. Rajavithi administration structured the campaign into broader facilities and service improvement initiatives, including the ventilation system. The time just after installation of the ventilation system was the most convenient for the survey and campaign launch. Survey results confirm or disconfirm the first hypothesis.

### Research Questions and Hypotheses

The first question the research addressed is, “What are the concentrations of bacteria, fungi, desflurane, and nitrous oxide in the Rajavithi Hospital PACU?” “Instruments” and “Sampling Environment and Procedures” sections provide details on measurement of waste gases and microorganisms. After measuring the levels of atmospheric pollutants, the hospital installed a new ventilation system. Installation immediately following measurement allows the hospital to witness the direct, positive effects of the ventilation system, which provides benefits to patients whose air quality stands to improve.

The second research question was “Does a HazCom and training program increase PACU worker attention to and concern for health and safety procedures?” The research answered this question with before-and-after surveys. The next three sections provide details of the instrument, participants, and sampling procedure. Following the completion of the informational campaign and second survey, measurements of atmospheric contaminants were taken a second time. Data was collected to confirm or disconfirm the first and second hypotheses.

Hypothesis 1: The new ventilation system decreases atmospheric pollution in the PACU.

Hypothesis 2: The hazard communication program increases perceived importance of health and safety procedures among PACU workers.

### Instruments

Rajavithi staff and administration created a simple survey during the planning stages of the campaign. A self-reporting survey assessed PACU workers’ attitudes toward health and safety procedures with a 5-point Likert scale where “1” was the lowest level of perceived importance and “5” was the highest. Hospital management administered the first survey in June 2016 and the second on April 25–30, 2017.

Waste anesthesia gas in the PACU atmosphere was measured using a TSI Velocicalc Multi-Function Ventilation Meter model 9565-P with a thermoanemometer articulated probe model 966. Atmospheric microorganism contamination was measured using a PBI International air sampler, model Duo SAS Super 360, with dishes for bacteria and fungi detection. The first measurements were taken in July–August 2016 prior to the campaign launch. A second sample was collected on April 25–30, 2017.

### Survey Participants

At the time of the study, there were 64 PACU staff members, all of whom were participants. Participation in the survey was mandatory as per employment agreement clauses pertaining to ongoing monitoring, supervision, and training. Participants included 5 males and 59 females, of which there were 61 anesthesia nurses and 3 anesthetists. Mean age of participants was 40. Roughly 60 percent of participants had more than 5 years of experience in their position. No patients were involved in the study.

### Sampling Environment and Procedures

Rajavithi Hospital (2018), in downtown Bangkok, is a 1,200-bed public medical center that can accommodate 40,000 in-patients and 1,000,000 out-patients annually. The hospital is staffed by more than 200 doctors, 800 nurses, and more than 4,000 support staff. The PACU is located adjacent to 22 operating rooms and has an area of 98.02 square meters. Surveys did not request individual respondents to identify themselves. All staff members were able to deposit completed questionnaires discretely and confidentially. No special permission or paperwork was required to collect the data.

Socially-desirable reporting is always a risk when using self-reporting surveys, however, any false or misleading response would have violated standards of professional ethics.

An outside firm collected air samples and tested for excess anesthetic gas and microorganisms using the instruments listed in “Instruments” section.

Following initial air sample and survey collections, PACU staff and hospital administration held an exploratory meeting in September 2016, during which time they developed an outline for a hazard communication and training program. The plan consisted of three parts: (1) commissioning a team to inspect the PACU for machine and structural flaws (i.e., broken doors, leaking water pipes, malfunctioning ventilation fans), (2) managing regular inspection of anesthetic equipment (i.e., calibration, leak inspection, joint and junction inspection, etc.), and (3) engaging PACU staff with an informational and training campaign.

### Ventilation System

During the communication and training program that followed the initial survey, Rajavithi Hospital installed one AirInSpace HEPA Guardian mobile ventilation system. Maximum air flow range of the system is up to 2,500 m^3^/hr. The Guardian system uses H14 and U15 filters.

### Communication and Training Campaign

Following the September 2016 open forum meeting among anesthesia nurses, anesthetists, and advisors on sterilization, the hospital initiated a HazCom program to increase the perceived importance of safety and health procedures among PACU workers and to decrease the levels of atmospheric contaminants in the PACU. The campaign commenced in December 2016 and ended in April 2017. The campaign had two main goals: to ensure all anesthesia equipment was effectively monitored and maintained, and to engage PACU staff with information regarding best-practices. A PACU management team was tasked with supervising equipment checks (i.e., calibration, cleaning, and leak inspection). Hospital administration collaborated with PACU senior staff members to engage workers with practical manuals, pamphlets, brochures, and informational media across multiple platforms. The hospital also initiated a PACU worker mentorship program and provided informational sessions with inside and outside experts.

## Results

Results showed an overall increase in perceived importance of health and safety procedures among PACU staff and decreased concentration of airborne contaminants in the PACU.

### Survey Data

Participants reported high perceived importance for all items on the survey in both stages of the study. Distribution data is reported in Table 1.

**Table 1:** Perceived Importance of Health and Safety Behaviors, before and after Campaign

Behavior	Before	After
	*M*(*SD*)	*M*(*SD*)
Hand washing after contact with patient	4.03(0.666)	4.09(0.495)
Wearing a mask when nursing a patient	4.16(0.623)	4.23(0.527)
Wearing an N95 mask when providing care to patients with respiratory diseases	4.14(1.111)	4.92(0.324)
Changing shoes when transferring patients outside the operating room	4.45(0.775)	4.84(0.366)
Changing shoes when entering the PACU	4.78(0.678)	4.91(0.294)
Ensuring patients’ mask are a suitable size for their face	4.83(0.380)	4.97(0.175)
Replacement of oxygen masks for PACU patients	4.97(0.175)	4.98(0.125)

Between the first and second survey, means increased and standard deviations decreased on all survey items. Thus, participants perceived all items to be of higher importance in the second survey compared to the first. Additionally, participants’ responses in the second survey were more uniform than in the first survey. When represented graphically, survey data were grouped more tightly around the high end of the scale in the second set as compared to the first.

### Airborne Pollutants in the PACU

Air samples revealed some contamination in the PACU. Moderate concentrations of N_2_O were found in both stages of the study. Low concentrations of desflurane were found. Extremely high levels of bacteria were found in the first measurement, a level which fell to just above normal in the second stage. Fungal contaminants were found in moderate amounts. Levels of all four contaminants were lower in the second stage as compared to the first (Table 2).

**Table 2:** Amount of Anesthetic Gas Residue, Bacteria, and Fungi in the PACU during Working Hours

Gas	Before	After	Recommended exposure limit
	*M*	*M*	
Nitrous oxide	21.86 ppm	20.47 ppm	25 ppm
Desflurane	0.25 ppm	0.21 ppm	2 ppm
Bacteria	>1,307 CFU/m^3^	182 CFU/m^3^	50–150 CFU/m^3^
Fungi	70.4 CFU/m^3^	35.8 CFU/m^3^	50–150 CFU/m^3^

## Discussion

Data collected answered the research question and confirmed both hypotheses.

Initial bacterial concentrations of 1,307 CFU/m^3^ exceeded all relevant guidelines the researcher discovered in review of literature (e.g., Fekadu & Getachewu, 2015; Napoli, Marcotrigiano, & Montagna, 2012; Park, Yeom, Lee, & Lee, 2013). Luksamijarulkul and Pipitsangjan (2015) considered 500 CFU/m^3^ as the ceiling for a safe hospital waiting room. Park et al. (2013) found bacterial and fungal pollutants in a Korean hospital lobby in lower concentration than this study found in the Rajavithi PACU at the beginning of the study. Fekadu and Getachewu (2015) considered levels above 1,000 CFU/m^3^ as “contaminated,” which is not a label a hospital desires to have upon its PACU. Hence, Rajavithi could not wait until after the HazCom and training were complete to install the ventilation system. In the second stage of the study, bacteria levels dropped by 86 percent and fungal count fell by roughly half as compared to initial measurements. Nitrous oxide and desflurane concentrations likewise fell between the first and second stage of measurements. Simultaneously, perceived importance of health and safety procedure among PACU workers increased between the first and second survey. Hospital administration inferred the ventilation installation and campaign were successful.

Due to the presence of an interfering factor (i.e., the ventilation system), it is impossible to determine if increases in perceived importance of behaviors among staff members directly led reduced contaminants in the PACU atmosphere. The hospital never intended the surveys to quantify a causal relationship; they were intended to show possible effects of the HazCom campaign. Hospital administration and PACU staff had no reason to suspect the ventilation system would not decrease the number of airborne contaminants in the PACU. The coincidental timing of the survey and ventilation system installation did not have effect on the significance of the study. If the hospital wishes to assess possible relationships between survey items or campaigns and air quality, another survey can be distributed. In fact, ongoing research on employee perceptions of the importance of protocols would likely benefit PACU and hospital staff.

Even though the campaign raised perceived levels of importance of protocols, hospital administration should remain vigilant that every member of the PACU staff always follows those health and safety procedures. Persons reading the results of this research should also remain aware of the limitations of self-reporting surveys, and keep in mind that perception of importance does not necessarily lead to behavior consistent with the item of perceived importance. Microorganism contamination levels in this study were higher than all recommended limits for hygienic spaces in the hospital environment, both before and after the campaign and ventilation installation. Whether these high contamination levels were due to practices among PACU workers, substandard anesthesia scavenging equipment, or still-insufficient ventilation is yet unknown. Considering the high level of risk posed by air pollution in PACUs, to both workers and patients, Rajavithi Hospital should continue its efforts to ensure best practices among employees, improve air quality through assessment of equipment, and ongoing testing of improved ventilation systems.

### Bacteria and Fungi

Ideally, every item on the survey should have received the maximum value for perceived importance. The survey item that received the lowest level of perceived importance in both stages was handwashing—a behavior that can serve to reduce the number of bacteria and fungi transferred from one area to another, both within the PACU and between the PACU and elsewhere in the hospital. Coincidentally, the behavior that participants reported second lowest perceived importance was wearing a mask when treating a patient—a behavior that can help prevent infection from airborne bacteria or fungi. Handwashing and use of antibacterial gels are essential sanitation and hygiene habits among workers in hospital generally, and especially in PACUs. While perceived importance is not an absolute indication of the likelihood that a worker engages in a behavior, hospital administration may consider this point to be the most significant finding in the study. Further reduction of contaminants may be available via more frequent hand sanitation.

Likewise concerning was the relatively large increase in perceived importance between surveys for items relating to changes of shoes during patient transfer, which suggested workers only became aware of its high importance, or overcame cognitive dissonance, after receiving consistent training and contact with information about the issue. Considering the relatively large increases in perceived importance for several other items on the survey, hospital administrators should investigate this item further.

Overall, concentration of bacteria fell from a seriously contaminated level to one just above RELs for a hygienic space in the hospital. The researcher inferred that, while worker attitudes probably played a positive role in this reduction, most of this change was due to installation of the ventilation system. Considering that concentrations at the end of the study were still on the high side, the researcher recommends hospital administration investigates installing additional ventilation while continuing to stress personal hygiene.

### Waste Anesthetic Gases

The item on the survey that showed the greatest increase between stages was regarding wearing the N95 mask when treating patients with respiratory diseases. This result demonstrated PACU workers’ concern for their health relating to potentially contagious diseases, but the lower levels of perceived importance of the other mask item may suggest workers are less concerned about potentially harmful effects of exposure to N_2_O and desflurane. While N_2_O levels were below established RELs, workers’ low perceived importance of facemasks in comparison to other survey items should receive some attention to ensure worker health and safety.

Little prior research was found regarding desflurane contamination, and this may be because desflurane is eliminated faster and therefore poses significantly less risk of toxicity due to long-term exposure when compared to older anesthetics like halothane (Edwards, 1999). Desflurane is one-eighths as potent as halothane, one-third as potent as sevoflurane, and one-fifth as potent as isoflurane (Eger, 1993). Desflurane concentrations at Rajavithi Hospital’s PACU were far below the hospital’s in-house RELs for halogenated agents of 2 ppm. One matter of concern for the research was that the hospital also uses sevoflurane in its ORs, but the administration did not approve monitoring and measurement of sevoflurane for the purposes of this study. Thus, even though desflurane levels are very low, it is still possible that hospital staff are exposed to harmful halogenated agents. Bearing that in mind, the researcher recommends Rajavithi Hospital should continue to monitor the PACU atmosphere for the detection and measurement of all anesthetic gases in use at the hospital, every six months as recommended by OSHA (2000).

### Continued Monitoring and Measurement at Rajavithi Hospital

Hospital administrators were, and should be, pleased to witness declines in airborne pollutants in the hospitals’ post anesthesia care unit. The campaign was an apparent success, especially with regards to bacteria, but the success was limited given that N_2_O levels were within 20 percent of RELs while bacterial and fungal concentrations still exceeded several sources’ RELs at the end of the study. The positive results of the study demonstrate that contaminations levels can be significantly reduced over the course of months with the addition of a ventilation system, and by making efforts to ensure all personnel involved remain conscious of the importance of health and safety procedures. Like all surgical hospitals, Rajavithi should continue to monitor and measure air quality in the PACU, troubleshoot potential impediments to further reduction of pollutants, and devise new solutions such that air quality is maintained well under RELs for all gases and particulate matter.

A main concern for hospital administration presently is to continue monitoring, and to do so frequently, so they can avoid making hasty or unwarranted conclusions about the effectiveness of the ventilation system and information campaign. One aspect of the study that may have been overlooked is that levels of airborne bacteria and fungi are naturally affected by seasonal changes (Aydogdu, Asan, Otkun, & Ture, 2005; Chaivisit et al., 2018; Frankel et al., 2012). Rajavithi administration should consider the possibility that the lower bacteria and fungi count in the second sample was due in part to ambient temperature and humidity. Notwithstanding the possibility that some of the variation was due to weather, research at Thai hospitals previously found that occupant number is the primary source of airborne bacteria and fungi (Chaivisit et al., 2018).

The single most important finding of this study was that microorganism contamination levels were higher than all recommended limits for hygienic spaces in a hospital environment. Whether these high contamination levels were due to practices among PACU workers, overcrowding in the hospital, substandard anesthesia scavenging equipment, or insufficient ventilation is yet unknown. Considering the high level of risk posed by air pollution in PACUs, to both workers and patients, Rajavithi Hospital should continue to assess equipment and ventilation systems. Hospital administration and donors may need to make room in the budget for new and improved gas scavenging and ventilation equipment.

### Broader Implications of the Study

Air quality is an issue of importance in all hospitals; and given that Rajavithi Hospital successfully implemented technology and an informational program which led to reduction in airborne pollutants in a hygienic zone, this research can be helpful to other hospitals in Thailand and elsewhere around the world. OSHA (2000) has promoted HEPA ventilation systems and HazCom programs for years, so the concepts are nothing new; but, by demonstrating their effectiveness in Thailand through this study, Thai hospital workers should be persuaded. Given that Rajavithi is a government hospital, the results of this study can easily be transmitted through the Ministry of Health to hundreds of other public hospitals. Dissemination of this research, in combination with continued research and monitoring, should further promote the issue of PACU hygiene and thereby improve recovery of patients, health and safety conditions for all persons in the PACU.

## Conclusion

This study measured the air quality in Rajavithi’s PACU in July 2016 and found concentrations of microorganisms, especially bacteria, were alarmingly high. Through installation of a ventilation system and implementation of a hazard awareness program, the hospital drastically reduced the concentration of airborne contaminants by March 2017. During the same period, an informational campaign aimed to increase employee awareness of safety protocols was undertaken. Two survey questionnaires showed PACU workers’ attitudes toward health and safety procedures improved over the course of the campaign. While the research cannot determine the exact extent to which the ventilation system or informational campaign led to reduction in PACU airborne contaminants, the overall result suggests both were successful.

The research led to recommendations for Rajavithi Hospital. First, the hospital should continue training and informing its PACU and other workers on the importance of all health and safety procedures. A compliance office may be of assistance in ensuring best practices. Second, the hospital should continue monitoring and measuring its air quality in the PACU, including testing for all anesthetic gases, and with the aim of identifying the specific strands of bacteria and fungi present. Also, the hospital should adjust the current ventilation system such that concentrations of pollutants fall within RELs, or the hospital should improve the ventilation system through additional purchases or remodeling of the PACU. Finally, the hospital should partner with officials from Ministry of Health and other hospitals to spearhead a campaign designed to help other hospitals in the Thai health system achieve proper hygiene in their PACU environments. Through continued research, monitoring, communication, and investment, both patients and hospital workers can avoid unnecessary complications and unfortunate consequences.

## Acknowledgments

The researcher expresses gratitude to Adam Tanielian for assistance in the editing and preparation of this manuscript.

## Declaration of Conflicting Interests

The authors declared no potential conflicts of interest concerning the research, authorship, or publication of this article.

## Appendix: Survey